# High quality draft genome sequence of *Corynebacterium ulceribovis* type strain IMMIB-L1395^T^ (DSM 45146^T^)

**DOI:** 10.1186/s40793-015-0036-7

**Published:** 2015-08-05

**Authors:** Atteyet F. Yassin, Alla Lapidus, James Han, T.B.K. Reddy, Marcel Huntemann, Amrita Pati, Natalia Ivanova, Victor Markowitz, Tanja Woyke, Hans-Peter Klenk, Nikos C. Kyrpides

**Affiliations:** Institut für Medizinische Mikrobiologie und Immunologie der Universität Bonn, Bonn, Germany; Theodosius Dobzhansky Center for Genome Bioinformatics, St. Petersburg State University, St. Petersburg, Russia; Algorithmic Biology Lab, St. Petersburg Academic University, St. Petersburg, Russia; Department of Energy Joint Genome Institute, Genome Biology Program, Walnut Creek, CA USA; Biological Data Management and Technology Center, Lawrence Berkeley National Laboratory, Berkeley, California USA; Leibniz Institute DSMZ – German Collection of Microorganisms and Cell Cultures, Braunschweig, Germany; Department of Biological Sciences, Faculty of Science, King Abdulaziz University, Jeddah, Saudi Arabia

**Keywords:** Gram-positive, Bovine skin, *Corynebacteriaceae*, *Actinobacteria*, GEBA

## Abstract

*Corynebacterium ulceribovis* strain IMMIB L-1395^T^ (= DSM 45146^T^) is an aerobic to facultative anaerobic, Gram-positive, non-spore-forming, non-motile rod-shaped bacterium that was isolated from the skin of the udder of a cow, in Schleswig Holstein, Germany. The cell wall of *C. ulceribovis* contains corynemycolic acids. The cellular fatty acids are those described for the genus *Corynebacterium*, but tuberculostearic acid is not present. Here we describe the features of *C. ulceribovis* strain IMMIB L-1395^T^, together with genome sequence information and its annotation. The 2,300,451 bp long genome containing 2,104 protein-coding genes and 54 RNA-encoding genes and is part of the Genomic Encyclopedia of Type Strains, Phase I: the one thousand microbial genomes (KMG) project.

**Electronic supplementary material:**

The online version of this article (doi:10.1186/s40793-015-0036-7) contains supplementary material, which is available to authorized users.

## Introduction

*Corynebacterium ulceribovis* IMMIB L-1395^T^ (= DSM 45146 = CCUG 55727) was first isolated from the skin of the udder of a cow with a profound ulceration [1]. The classification and identification of this species was based on chemotaxonomic traits and biochemical tests, which were supplemented by 16S rRNA gene phylogentic assessments. Since then, there have been neither reported cases associating strains of *C. ulceribovis* with animal infections nor has there been documented cases of its isolation in humans. Although members of the genus *Corynebacterium* are generally regarded as commensal skin colonizer in humans and animals, e.g. *Corynebacterium amycolatum*, *Corynebacterium bovis*, *Corynebacterium mastitidis*, *Corynebacterium pseudotuberculosis*, *Corynebacterium xerosis* and *Corynebacterium ulcerans* [2–4], the question remains unanswered whether to consider *C. ulceribovis* as belonging to the resident or transient microbes of bovine skin. Therefore, the veterinary medical importance of *C. ulcerobovis* is unclear and remains to be assessed.

Here we present a summary classification and a set of features for *C. ulceribovis* IMMIB-L1395^T^ together with the description of the complete genomic sequencing and annotation of DSM 45146^T^ providing insights into candidate genes involved in some basic biological processes.

## Organism information

### Classification and features

Following the published hierarchial classification of *Actinobacteria* [5, 6], *C. ulceribovis* belongs to the genus *Corynebacterium* of the family *Corynebacteriaceae*, one of six suprageneric taxa included in the suborder *Corynebacterineae* of the order *Actinomycetales* of the subclass *Actinobacteridae* of the class *Actinobacteria*.

#### Morphology and physiology

**Fig. 1:**
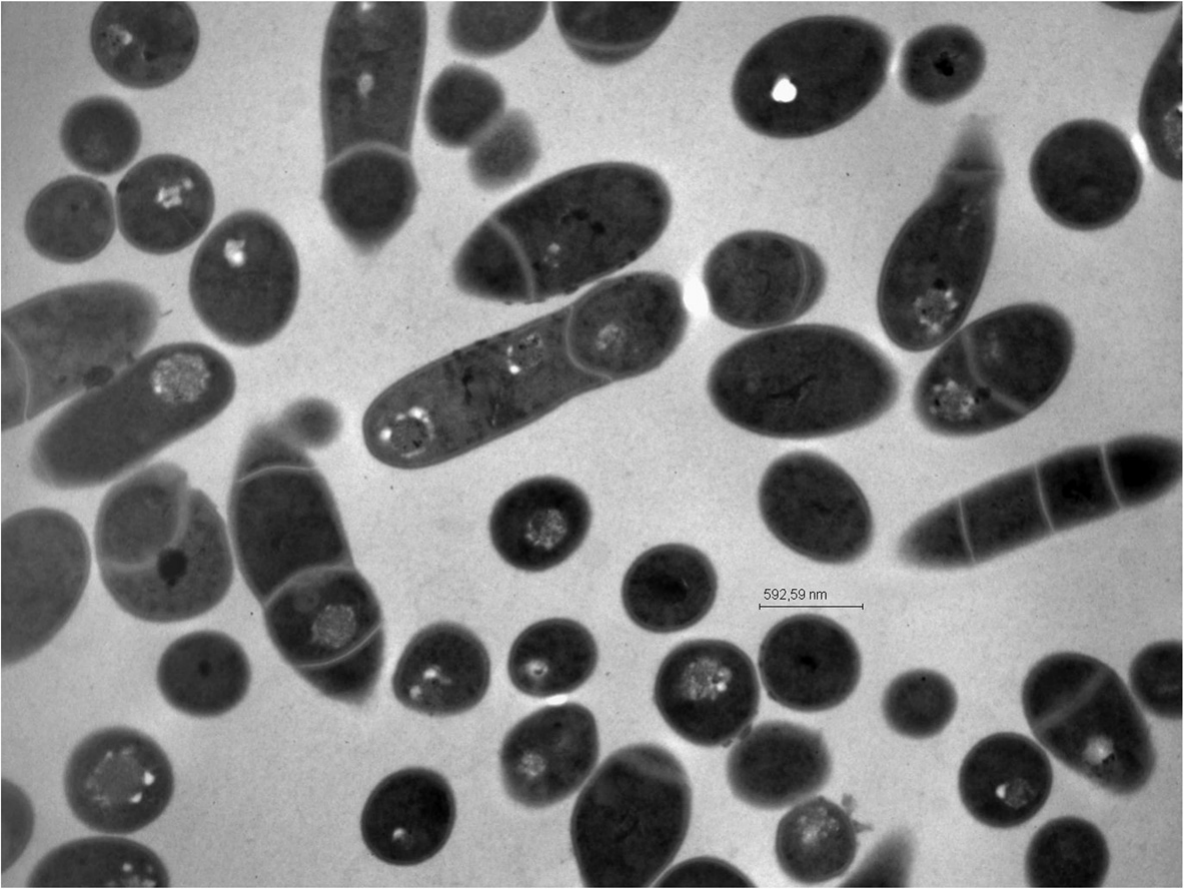
Transmission electron micrograph showing cells of *C. ulceribovis* IMMIB L-1395^T^. Cells are rod-shaped, which upon extended incubation, divide in spherical form. The cocci are arranged singly

Surface colonies of *C. ulceribovis* IMMIB L-1395^T^ grown on Columbia blood agar supplemented with 5 % sheep blood (BD; Beckton, Dickenson) are circular (2.0-4.0 mm in diameter), gray, opaque, non-hemolytic and entire edged after 48 h of incubation at 37 °C in a 5 % CO_2_ atmosphere. Cells are Gram-stain positive, nonmotile and non-spore-forming. The cells appear as slender, irregular rods (0.5 × 1.16 μm), which upon extended incubation become granular and segmented and resemble small irregular cocci (0.59 μm in diameter). The cocci are usually arranged singly (Fig. 1). Optimum growth temperature is 37 °C. The organism is facultatively anaerobic and catalase-positive. Nitrate is not reduced to nitrite, gelatin is not liquified and esculin hydrolysis is negative. Hippurate and Tween 80 are hydrolysed. Acid and alkaline phosphatases, esterase lipase (C8), leucine arylamidase, pyrazinamidase and naphthol-AS-BI-phosphohydrolase are detected in the API ZYM (bioMériux) gallery, while no activity is detected for arginine dihydrolase, chymotrypsin, cysteine arylamidase, esterase (C4), α-fucosidase, α-galactosidase, β-galactosidase, β-glucuronidase, α-glucosidase, β-glucosidase, N-acetyl-β-glucosaminidase, lipase (C14), α-mannosidase, pyrrolidonyl arylamidase, trypsin or valine arylamidase. The organism is susceptible to ampicillin (2 μg), penicillin (1 unit), imipenem (10 μg), ciprofloxacin (5 μg), moxifloxacin (5 μg), cefoxitin (30 μg), gentamicin (10 μg), clindamycin (2 μg), erythromycin (15 μg), rifampicin (5 μg), tigecycline (15 μg) and vancomycin (5 μg); all data from [1]. A summary of the classification and general features of strain IMMIB L-1395^T^ is presented in Table 1.

**Table 1:** Classification and general features of *C. ulceribovis* IMMIB L-1395^T^ in accordance with the MIGS recommendations [127] published by the Genomic Standards Consortium [128]

MIGS ID	Property	Term	Evidence code
	Current classification	Domain *Bacteria*	TAS [129]
		Phylum *Actinobacteria*	TAS [5]
		Class *Actinobacteria*	TAS [5]
		Order *Actinomycetales*	TAS [5]
		Family *Corynebacteriaceae*	TAS [6]
		Genus *Corynebacterium*	TAS [130]
		Species *Corynebacteriu ulceribovis*	TAS [1]
		Type strain : IMMIB-L1395T	TAS [1]
	Gram stain	Positive	TAS [1]
	Cell shape	Rod	TAS [1]
	Motility	Non-motile	TAS [1]
	Sporulation	Non-sporulating	TAS [1]
	Temperature range	Mesophile	TAS [1]
	Optimum temperature	37 °C	TAS [1]
	pH Range; Optimum	6.8-8.0; 7.3	TAS [1]
	Carbon source	Glucose	TAS [1]
	Energy source	Chemoorganotroph	TAS [1]
MIGS-6	Habitat	Skin, Host	TAS [1]
MIGS-6.3	Salinity	Not reported	
MIGS-22	Oxygen requirement	Aerobic-facultative anaerobic	TAS [1]
MIGS-15	Biotic relationship	Free living	TAS [1]
MIGS-14	Pathogenicity	Unknown	NAS
MIGS-4	Geographic location	Schleswig Holstein, Germany	TAS [1]
MIGS-5	Sample collection date	1996	TAS [1]
MIGS-4.1	Latitude	9.588	IDA, TAS [1]
MIGS-4.2	Longitude	54.209	IDA, TAS [1]
MIGS-4.3	Depth	Not recorded	
MIGS-4.4	Altitude	Not recorded	

Evidence codes – IDA: Inferred from Direct Assay; TAS: Traceable Author Statement (i.e., a direct report exists in the literature); NAS: Non-traceable Author Statement (i.e., not directly observed for the living, isolated sample, but based on a generally accepted property for the species, or anecdotal evidence). These evidence codes are from the Gene Ontology project [126]

#### Chemotaxonomy

*C. ulceribovis* has cell-wall chemotype IV, which includes the presence of *meso*-diaminopimelate (*meso*-DAP), arabinose and galacose. Corynemycolic acids are present. The major cellular fatty acids are palmitic (C_16 : 0_) and oleic (C_18 : 1_ω9c) acids, which constitute more than 95 % of the total fatty acids content. Tuberculostearic acid is not present [1]. The G + C content calculated from the genome draft sequence is 59.2 mol%. No information is available on the poar lipid or respiratory lipoquinone composition.

#### 16S rRNA gene analysis and phylogeny

Phylogenetic analyses were performed using the ARB-package [7]. Evolutionary distances were calculated using the Jukes-Cantor method [8]. Phylogenetic trees were generated by maximum-parsimony (ARB_PARS), neighbour-joining and maximum-likelihood (RAxML; [9]) facilities as implemented in the ARB package. Topologies of the neighbour-joining tree were evaluated using bootstrap analyses [10] based on 500 resamplings. The sequence of the single 16S rRNA gene copy (1397 nucleotides) in the genome of *C. ulceribovis* DSM 45146^T^ was added to the ARB database [7] and compared with the 16S rRNA gene sequences of the type strains of *Corynebacterium* species obtained from the NCBI database. This sequence does not differ from the previously published 16S rRNA sequence (AM922112). The highest-scoring sequence of a neighboring species was (HE983829) reported for the type strain of *C. lactis* DSM 45799^T^, which showed a similarity of 96.5 %.

**Fig. 2:**
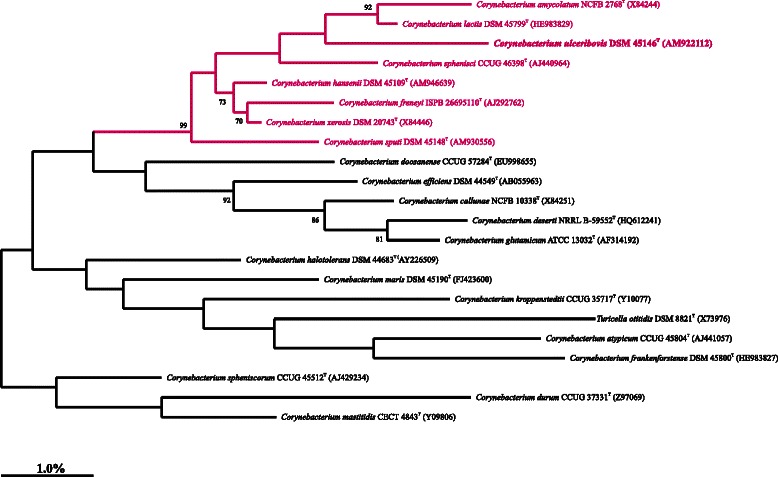
Maximum likelihood phylogenetic tree based on 16S rRNA gene sequences highlighting the position of *C. ulceribovis* DSM 45146^T^ relative to the type strains of other *Corynebacterium* species. Numbers at the nodes are support values from 500 bootstrap replicates of >70 %. Bar, 1.0 % sequence divergence. The tree was generated using the ARB software package [7]

Figure 2 shows the phylogenetic position of *C. ulceribovis* DSM 45146^T^ within the genus *Corynebacterium* in a 16S rRNA based tree. It is evident from the tree that *C. ulceribovis* DSM 45146^T^ together with *C. amycolatum*, *C. lactis*, *C sphenisci*, *C. sputi*, *C. hansenii*, *C. freneyi* and *C. xerosis* constitute a distinct monophyletic group within the genus *Corynebacterium*. The clustering of this group of species was also observed in recent study of the phylogeny of the 16S rRNA gene in *Actinobacteria* [11]. The coherency of members of this clade was strongly supported by 99 % bootstrap value and by sharing a distinct set of 16S rRNA signature nucleotides at positions: 131–231 (C-G) and 1308–1329 (C-G). At these positions all other *Corynebactium* species contain the pairs (U-A). Members of this subclade showed high 16S rRNA gene sequence similarities ranged between 95.3 % and 99.5 %.

To further study the phylogenetic relationship between *C. ulceribovis* and the type strains of some members of this subcluster such as *C. freneyi* and *C. sputi*, whose genome sequences are available, we compared homologous proteins annotated as polyketide synthase (Pks13), fatty acid CoA ligase (FadD32), trehalose corynomycolyl transferase (CmtC) and acetyl coA carboxylase (AccD3), enzymes which form an integral part of the mycolic acid biosynthetic pathway. BLASTP analysis showed that the average amino acid identity between homologous pairs from *C. ulceribovis**,**C. freneyi* and *C. sputi* was around 79 % for AccD3, 62 % for Pks13, 63 % for FadD32 and 49 % for CmtC. The phylogenetic trees constructed using the maximum likelihood and neighbor-joining methods based on this data set of protein sequences showed that *C. ulceribovis*, *C. freneyi* and *C. sputi* clustered adjacent to each other within the genus *Corynebacterium* (data not shown). Thus, one may hypothesize that this monophyletic group deserves to be recognized as the core of a new genus. However, expanded datasets are needed to affirm the phylogenetic relationship between members of this clade and better resolve the intrageneric relationship between them. In addition, further study will be required to identify synapomorphies to delineate this lineage before a taxonomic conclusion can be made.

## Genome sequencing and annotation

### Genome project history

The strain was selected for sequencing on the basis of its phylogenetic position [12, 13], and is part of the Genomic Encyclopedia of Type Strains, Phase I: the one thousand microbial genomes (KMG) project [14], a follow-up of the Genomic Encyclopedia of *Bacteria* and *Archaea* (GEBA) pilot project [15], which aims at increasing the sequencing coverage of key reference microbial genomes and to generate a large genomic basis for the discovery of genes encoding novel enzymes [16]. KMG-I is the first of the production phases of the “Genomic Encyclopedia of *Bacteria* and *Archaea*: sequencing a myriad of type strains initiative and a Genomic Standards Consortium project [17]. The genome project is deposited in the Genomes On Line Database [18] and the genome sequence is available from GenBank. Sequencing, finishing and annotation were performed by the DOE Joint Genome Institute (JGI) using state of the art sequencing technology [19]. A summary of the project information is presented in Table 2.

**Table 2:** Project information

MIGS ID	Property	Term
MIGS-31	Finishing quality	High-Guality Draft [131]
MIGS-28	Libraries used	Illumina STD shotgun library
MIGS-29	Sequencing platforms	Illumina HiSeq2000
MIGS-31.2	Fold coverage	123.8 ×
MIGS-30	Assemblers	Velvet (version 1.1.04), ALLPATHS v. r41043
MIGS-32	Gene calling method	Prodigal 2.5
	Locus Tag	A3EC
	Genbank ID	AQUY00000000
	Genbank Date of Release	April 19, 2013
	GOLD ID	Gp0013740
	BIOPROJECT	PRJN165381
MIGS-13	Source material identifier	DSM 45146^T^
	Project relevance	Tree of Life, GEBA-KMG

### Growth conditions and genomic DNA preparation

*C. ulceribovis* DSM 45146^T^, was grown in DSMZ medium 535a (Trypticase Soy Broth Agar + Blood) [20] at 37 °C. Genomic DNA was isolated using MasterPure Gram Positive DNA Purification Kit (Epicentre MGP04100) following the standard protocol provided by the manufacturer with the following modifications for the cell lysis solution: additional digest with 1 μl proteinase K (50 μg/μl), 7.5 μl achromopetidase (1 U/μl), 7.5 μl lysostaphin (1 U/μl), 3 μl lysozym (700 U/μl) and 7.5 μl mutanolysin (1 U/μl). Protein precipitation with 200 μl protein precipitation buffer (PPT) and incubation on ice over night followed by incubation (60 min, 37 °C) with 50 μl proteinase K. DNA is available through the DNA Bank Network [21].

### Genome sequencing and assembly

The draft genome of *C. ulceribovis* DSM 45146^T^ was generated using the Illumina technology [22]. An Illumina Std shotgun library was constructed and sequenced using the Illumina HiSeq 2000 platform which generated 17,830,172 reads totaling 2,674.5 Mbp. All general aspects of library construction and sequencing performed at the JGI can be found at [23]. All raw Illumina sequence data was passed through DUK, a filtering program developed at JGI, which removes known Illumina sequencing and library preparation artifacts [24]. The following steps were then performed for assembly: (1) filtered Illumina reads were assembled using Velvet (version 1.1.04) [25], (2) 1–3 Kbp simulated paired end reads were created from Velvet contigs using wgsim [26], (3) Illumina reads were assembled with simulated read pairs using Allpaths–LG (version r41043) [27]. Parameters for assembly steps were: 1) Velvet (velveth: 63 –shortPaired and velvetg: −very clean yes –export-Filtered yes –min contig lgth 500 –scaffolding no –cov cutoff 10) 2) wgsim (−e 0 –1 100 –2 100 –r 0 –R 0 –X 0) 3) Allpaths–LG (PrepareAllpathsInputs: PHRED 64 = 1 PLOIDY = 1 FRAG COVERAGE = 125 JUMP COVERAGE = 25 LONG JUMP COV = 50, RunAllpathsLG: THREADS = 8 RUN = std shredpairs TARGETS = standard VAPI WARN ONLY = True OVERWRITE = True). The final draft assembly contained 8 contigs in 8 scaffolds. The total size of the genome is 2.3 Mbp and the final assembly is based on 284.2 Mbp of Illumina data, which provides an average 123.8 × coverage of the genome.

### Genome annotation

Genes were identified using Prodigal [28] as part of the DOE-JGI Annotation pipeline [29] followed by a round of manual curation using the JGI GenePRIMP pipeline [30]. The predicted CDSs were translated and used to search the National Center for Biotechnology Information non-redundant database, UniProt, TIGRFam, Pfam, PRIAM, KEGG, COG, and InterPro databases. Additional gene prediction analysis and functional annotation was performed within the Integrated Microbial Genomes [31].

## Genome properties

**Fig. 3:**
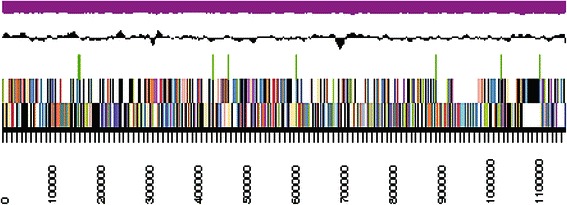
Graphical map of the largest scaffold of the genome. From bottom to the top: Genes on forward strand (color by COG categories), Genes on reverse strand (color by COG categories), RNA genes (tRNA green, rRNA red, other RNAs black), GC content, GC skew (purple/olive)

The assembly of the draft genome sequence consists of eight scaffolds amounting to a 2,300,451 bp long chromosome with a GC content of approximately 59.2 % (Table 3 and Fig. 3). Of the 2,158 genes predicted, 2,104 were protein encoding and 54 RNA encoding genes. Within the genome, 22 pseudogenes were also identified. The majority of genes (73.45 %) were assigned a putative function whilst the remaining genes were annotated as hypothetical proteins. The distribution of genes into COGs functional categories is presented in Table 4.

**Table 3:** Genome statistics

Attribute	Value	% of total
Genome size (bp)	2,300,451	100.00
DNA coding region (bp)	2,121,258	92.21
DNA G + C content (bp)	1,362,599	59.23
DNA scaffolds	8	
Total genes	2,158	100.00
Protein-coding genes	2,104	97.50
RNA genes	54	2.50
Pseudo genes	22	1.02
Genes in internal clusters	153	7.09
Genes with function prediction (proteins)	1,585	73.45
Genes assigned to COGs	1,368	63.39
Genes with Pfam domains	1,693	78.45
Genes with signal peptides	133	6.16
Genes with transmembrane helices	541	25.07
CRISPR repeats	2

**Table 4:** Number of genes associated with general COG functional categories

Code	Value	% of total^a^	Description
J	161	10.76	Translation, ribosomal, structure and biogenesis
A	1	0.07	RNA processing and modification
K	82	5.48	Transcription
L	84	5.61	Replication, recombination and repair
B	1	0.07	Chromatin structure and dynamics
D	21	1.4	Cell cycle control, Cell division; Chromosome partitioning
V	34	2.27	Defense mechanisms
T	30	3.01	Signal transduction mechanisms
M	72	4.81	Cell wall/membrane biogenesis
N	4	0.27	Cell motility
U	19	1.27	Intracellular trafficking and secretion
O	69	4.61	Posttranslational modification, protein turnover, chaperones
C	99	6.62	Energy production conversion
G	100	6.68	Carbohydrate transport and metabolism
E	140	9.36	Amino acid transport metabolism
F	66	4.41	Nucleotide transport and metabolism
H	99	6.62	Coenzyme transport and metabolism
I	95	6.35	Lipid transport and metabolism
P	98	6.55	Inorganic ion transport and metabolism
Q	30	2.01	Secondary metabolite biosynthesis, transport and catabolism
R	109	7.29	General function prediction only
S	61	4.08	Function unknown
-	790	36.61	Not in COGs

^a^The total is based on the total number of protein-coding genes in the genome

## Insights from the genome sequence

### Insights into carbohydrate metabolism

As mentioned previously glucose was the primary carbohydrate utilized by *C. ulceribovis*. This sugar is likely to be imported into the cells by a homolog of the phosphoenolpyruvate (PEP): sugar phosphotransferase system (PTS), which is responsible for the transport and concomitant phosphorylation of various sugars across the cell membrane. Exploring the genome of *C. ulceribovis* revealed the presence of the genes encoding for the PTS proteins. These include the gene *ptsI* encoding for enzyme I ([EI], A3ECDRAFT_1792) and the gene *ptsH* encoding for the histidine carrier protein ([HPr], A3ECDRAFT_1795), as well as the gene *ptsG* encoding for the glucose-specific enzyme II ([EII^Glc^], A3ECDRAFT_1683) and the gene *ptsFru* encoding for the fructose-specific enzyme II ([EII^Fru^], A3ECDRAFT_1794). A single copy of each of these genes was found within the genome of *C. ulceribovis*. The EI and HPr proteins lack sugar specificity and catalyze the transfer of phosphoryl groups from PEP to EIIs. EIIs are complex enzymes consisted of three protein domains, namely, IIA, IIB and IIC. IIA and IIB are phosphoryl transfer proteins of the PTS, whereas IIC is the actual sugar permease [32, 33]. The presence of the *ptsG* gene confirmed the ability of this organism to utilize glucose as source of carbon and energy.

Besides the PTS, the genome of *C. ulceribovis* contains a set of genes predicted to encode a carbohydrate ABC transporter (A3ECDRAFT_0345 to A3ECDRAFT_0348), which belongs to the CUT1 family (TC 3.A.1.1-). This ABC transporter composed of two homologous genes encoding two permeases (A3ECDRAFT_0345 and A3ECDRAFT_0346), one encoding a substrate-binding protein (A3ECDRAFT_0347) and one encoding ATP-binding protein (A3ECDRAFT_0348). Members of the CUT1 family are known to transport diverse di- and oligosaccharides, glycerol, glycerol-phosphate and polyols [34]. However, the sugar transported by this ABC transporter remains to be determined in *C. ulceribovis*. The genes encoding this ABC transporter are located downstream from the genes encoding a two component system consisting of a sensor histidine kinase and a response regulator.

#### Central carbohydrate metabolism

The genes envolved in metabolic pathways were analyzed in detail using the information present in KEGG database [35]. It is apparent from inspection of the genome sequence of *C. ulceribovis* that the genome contains a complete set of genes coding for the enzymes of the central carbohydrate metabolism, including those that are used in glycolysis, gluconeogenesis, the pentose phosphate pathway (PPP) and the tricarboxylic acid cycle (TCA). The glycolytic enzymes catalyzing the three irreversible steps of glycolysis, glucokinase GCK ([EC:2.7.1.2]; A3ECDRAFT_1543), phosphofructokinase PFK ([EC:2.7.1.11]; A3ECDRAFT_0613) and pyruvate kinase PK ([EC:2.7.1.40], A3ECDRAFT_1959 and A3ECDRAFT_1661), were identified. The key gluconeogenic enzymes phosphoenolpyruvate carboxykinase PEP CK ([EC:4.1.1.32]; A3ECDRAFT_1920), which catalyzes the conversion of oxaloacetate to PEP; glyceraldehyde-3-phosphate dehydrogenase GAPDH ([EC:1.2.1.12]; A3ECDRAFT_0423 and A3ECDRAFT_0908), which catalyzes the conversion of 1,3-biphosphoglycerate to glyceraldehyde-3-phosphate; and fructose-1,6-biphosphatase GlpX ([EC:3.1.3.11]; A3ECDRAFT_0476), which catalyzes the hydrolysis of fructose 1,6-bisphosphate to fructose 6-phosphate and inorganic phosphate, were identified. The emzymes of the oxidative (OPP) and nonoxidative branches of the pentose phosphate pathway were identified. The three enzymes, glucose-6-phosphate 1-dehydrogenase G6PDH ([EC:1.1.1.49]; A3ECDRAFT_0915), 6-phosphogluconolactonase 6PGL ([EC:3.1.1.31]; A3ECDRAFT_0913) and 6-phosphogluconate dehydrogenase (6PGDH) ([EC:1.1.1.44]; A3ECDRAFT_1067), which catalyze the three irreversible reractions of OPP branch were present. The primary enzymes that mediate the reversible reactions of the non oxidative PPP branch, transketolase TKT ([EC:2.2.1.1]; A3ECDRAFT_0917) and transaldolase TALDO ([EC:2.2.1.2]; A3ECDRAFT_0916), were also present.

A set of genes encoding enzymes necessary to drive a complete oxidative tricarboxylic acid cycle were found in the genome of *C. ulceribovis*. These include genes encoding citrate synthase [EC:2.3.3.1] (*gltA*, A3ECDRAFT_1136), aconitase [EC:4.2.1.3] (*acnA*, A3ECDRAFT_0939), isocitrate dehydrogenase [EC:1.1.1.42] (*icd*, A3ECDRAFT_0312), α-ketoglutarate dehydrogenase [EC:2.3.1.61] (*sucB*, A3ECDRAFT_1518), succinyl-CoA:acetate CoA-transferase [EC:3.8.3.18] (*cat1*, A3ECDRAFT_0443 and A3ECDRAFT_0629), succinate dehydrogenase [EC:1.3.5.1] (*sdhAB*, A3ECDRAFT_0086 and A3ECDRAFT_0087), fumarate hydatase [EC:4.2.1.2] (*fumC*, A3ECDRAFT_0475), NAD-dependent malate dehydrogenase [EC:1.1.1.37] (*mdh*, A3ECDRAFT_1397) and FAD-dependent malate:quinone oxidoreductase [EC:1.1.5.4] (*mqo*, A3ECDRAFT_0727). The gene encoding for the anaplerotic enzyme phosphoenolpyruvate carboxylase ([EC:4.1.1.31]; A3ECDRAFT_0911), which catalyzes the synthesis of oxaloacetate from pyruvate was also identified. Genes coding for the enzymes of the anaplerotic glyoxylate cycle, isocitrate lyase (EC:4.1.3.1; A3ECDRAFT_0081) and malate synthase ([EC:2.3.3.9], A3ECDRAFT_0082), were present in the genome.

#### Glycogen metabolism

Glycogen, a soluble α-linked glucose polymer (or α-glucan) with ~90 % α-1,4-links in its backbone and ~10 % α-1,6-linked branches, is a source of carbon and energy storage in a wide variety of organisms, including bacteria [36]. Inspection of the genome revealed that *C. ulceribovis* was equipped with the genes encoding proteins envolved in glycogen biosynthesis by the classical GlgC/GlgA and the GlgE pathways. Key genes encoding enzymes involved in the GlgC/GlgA pathway include: *glgC*, encoding for glucose-1-phosphate adenyltransferase GlgC ([EC:2.7.7.27], A3ECDRAFT_0532) which catalyzes the production of ADP-glucose from ATP and glucose-1-phosphate; *glgA*, encoding for glycogen synthase GlcA ([EC:2.4.1.21], A3ECDRAFT_0531) which catalyzes the successive addition of glucose from the glycosyl-nucleotide to the growing α-1,4-linked chain to generate the linear glucan; and *glgB*, encoding for the α-1,4-glucan branching enzyme GlgB ([EC:2.4.1.18], A3ECDRAFT_0591) which catalyzes the formation of α-1,6-glucosidic linkages in the linear α-1,4-glucans to produce glycogen.

In case of the GlgE pathway, three of the four genes (*treS*, *pep2*, *glgE* and *glgB*) encoding for the enzymes involved in this pathway were found: the *glgE* gene encoding for maltosyl transferase GlgE ([EC:2.4.99.16], A3ECDRAFT_0592), the *pep2* gene encoding a maltokinase Pep2 ([EC2.7.1.175], A3ECDRAFT_1428) and the previously mentioned *glgB* gene encoding for the GlgB ([EC:2.4.1.18], A3ECDRAFT_0591). BLASTP analysis revealed that the Pep2 protein is a maltokinase which forms a complex with trehalose synthase TreS. This is not surprising partly due to the fact that the *pep2* (also called *mak*) gene is usually linked with the *treS* gene and in some micro-organisms like *Psuedomonas entamophila*, *Rubrobacter xylanophilus* and in numerous members of the class *Actinobacteria* the two genes are fused into a single gene [37–39]. The GlgE pathway requires trehalose as a precursor of α-glucan synthesis using the combined action of the four enzymes [37, 40–42]. In this pathway, trehalose is first isomerized to maltose by trehalose synthase (TreS). Next, maltose is phosphorylated to maltose-1-phosphate by maltose kinase (Pep2) by expending a molecule of ATP. The phospho-activated disaccharide is a substrate for maltosyltransferase (GlgE). GlgE uses maltose-1-phosphate to elongate α(1 → 4) linked glucan chains. GlgB, the last enzyme of this pathway, mediates α(1 → 6)-branching of the glucan chain [43].

In addition to the gene involved in glycogen biosynthesis two genes, *glgP* and *glgX*, which encode for glycogen phosphorylase GlgP ([EC:2.4.1.1], A3ECDRAFT_1662) and the glycogen debranching enzyme GlgX ([EC:3.2.1_], A3ECDRAFT_1629), respectively, were identified in the genome. These enzymes mediate glycogen degradation. GlgP catalyzes the sequential phosphorolysis of glycogen to release glucose-1-phosphate, whereas the enzyme GlgX acts by remodeling of the glycogen branches to permit further degradation.

#### Trehalose metabolism

Trehalose is a disaccharide composed of two glucose units which are linked in an α, α-1,1-glycosidic linkage. It is an energy store and a stress-protectant, helping bacteria to survive desiccation, cold and osmotic stress [44]. Trehalose is also an integral component of cell wall trehalose dimycolates (TDM, cord factor) found in species of the genera *Mycobacterium*, *Nocardia*, *Rhodococcus* and *Corynebacterium* [45, 46]. Inspection of *C. ulceribovis* genome revealed the presence of genes encoding for proteins envolved in trehalose biosynthesis via the GalU-OtsA-OtsB and the TreY-TreZ pathways. The GalU-OtsA-OtsB pathway is catalyzed by the *galU*, *otsA* and *otsB* gene products, including the enzymes UTP--glucose-1-phosphate uridyltransferase GalU ([EC:2.7.7.9], A3ECDRAFT_1217), trehalose-6-phosphate synthase OtsA ([EC:2.4.1.15], A3ECDRAFT_1788) and trehalose-6-phosphate phosphatase OtsB ([EC:3.1.3.12], A3ECDRAFT_1791), respectively, whilst the TreY-TreZ pathway is catalyzed by the *treY* and *treZ* gene products, which include the enzymes maltooligosyltrehalose synthase TreY ([EC:5.4.99.15], A3ECDRAFT_1625) and maltooligosyltrehalose trehalohydrolase TreZ ([EC:3.2.1.141], A3ECDRAFT_1615), respectively. The GalU-OtsA-OtsB pathway involves trehalose synthesis from UDP-glucose and glucose-6-phosphate [47, 48], whereas the TreY-TreZ pathway involves trehalose biosynthesis from glycogen-like α(1 → 4)-linked glucose polymers [47, 49].

Additionally, examination of the genome revealed the presence of a gene encoding for trehalose phosphorylase ([EC:2.4.1.64], A3ECDRAFT_0084). This enzyme catalyzes the phosphorolysis of trehalose to produce glucose-1-phosphate and glucose. This reaction is reversible and could give rise to trehalose from glucose-1-P and glucose [50].

### Insight into lipids metabolism

#### Fatty acid biosynthesis

Fatty acids biosynthesis is mediated by enzymes catalyzing several iterative cycles of reaction steps including condensation, reduction, dehydration and reduction [51, 52]. The genes encoding for enzymes necessary for fatty acid biosynthesis in *C. ulceribovis* DSM 45146^T^ were identified. Inspection of the genome revealed the presence of a single *fas1* gene encoding type I fatty acid synthase FAS I ([EC:2.3.1.-], A3ECDRAFT_2083). BLASTP analysis revealed that FAS I (A3ECDRAFT_2083) was identical to homologs (NCgl0802) in *C. glutamicum* ATCC13032^T^ and (HMPREF0281_00958) in *C. ammoniagens* DSM 20306^T^ sharing 53 % and 52 % identities, respectively. FAS I (A3ECDRAFT_2083) is a single polypeptide of 3055 amino acid residues, which contained all the catalytic domains necessary to perform the iterative series of reactions for *de novo* fatty acids synthesis. The individual component enzymes of the various catalytic domains are acyl transferase (AT), enoyl reductase (ER), β-hydroxyacyl dehydratase (DH), malonyl/palmitoyl transferase (MPT), acyl carrier protein (ACP), β-ketoacyl reductase (KR), and β-ketoacyl synthase (KS) [53].

In addition to the *fas1* gene, genes encoding for the putative subunits of acetyl-CoA carboxylase were found: one gene encoding for biotin carboxylase BC (α subunit) ([EC:6.3.4.14], A3ECDRAFT_2085) and the other encoding for carboxyltransferase CT (β subunit) (A3ECDRAFT_2084). Acetyl-CoA carboxylase catalyzes the biotin-dependent carboxylation of acetyl-CoA to produce malonyl-CoA in the first committed step of the fatty acid biosynthesis pathway. Malonyl-CoA is then made available to be utilized by the multifunctional type I FAS for *de novo* biosynthesis of fatty acids. FAS I synthesizes both saturated (C_16:0_ and C_18:0_) and monounsaturated (C_18:1_ω9c) fatty acids [54]. In *C. ulceribovis* the results of cellular fatty acids analysis are in agreement with the functional characteristics of FAS I.

#### Fatty acid catabolism

For the catabolism of fatty acids, 16 genes encoding for proteins predicted to be involved in the β-oxidation pathway of fatty acid degradation were identified. These include: four *fadE* genes encoding for acyl-CoA dehydrogenase ([EC:1.3.8.7], A3ECDRAFT_0464, _1504, _1084, _1608, _1609), two *fadD* genes encoding for fatty acid CoA ligase ([EC:6.2.1.3], A3ECDRAFT-0100 and A3ECDRAFT_1435), one *fadJ* gene encoding for 3-hydroxyacyl-CoA dehydrogenase ([EC:1.1.1.35], A3ECDRAFT_1916), three *fadA* genes encoding for acetyl-CoA acyltransferase ([EC:2.3.1.9], A3ECDRAFT_1199, _1765, _1915) and four *echA* genes encoding for enoyl-CoA hydratase ([4.2.1.17], A3ECDRAFT_1142, _1525,_2066, _2067). In addition, one *acx* gene encoding for acyl-COA oxidase ACOX1 ([EC:1.3.3.6], A3ECDRAFT_2105), which catalyzes the desaturation of fatty acyl-CoA thioesters and donates electrons directly to molecular oxygen generating H_2_O_2_ [55, 56]. The subsequent detoxification of the resulting H_2_O_2_ is catalyzed by catalase ([EC:1.11.1.6]; A3ECDRAFT_0111) encoded by the *katA* gene of *C. ulceribovis*. The existence of considerable set of genes putatively involved in β-oxidation, suggested the ability of *C. ulceribovis* to mobilize the energy and carbon stored in fatty acids with different chain-lengths.

#### Corynomycolic acid biosynthesis and processing

Mycolic acids, long-chain α-alkyl, β-hydroxy fatty acids, are major components of the cell wall of several genera of *Corynebacterineae*. They are found either covalently linked to the cell wall arabinogalactan, to form mycolyl arabinogalactan, or acylated to trehalose units to form trehalose monomycolate (TMM) and trehalose dimycolate (TDM) [57–59]. Mycolic acids covalently linked to the cell wall form a hydrophobic permeability barrier, also referred to as the mycomembrane, which contributes to the low permeability of the envelope of *Corynebacterineae* and the natural resistance of these microorganisms to various antibiotics [45, 60, 61]. Mycolic acids vary in size and complexity within the different genera of *Corynebacterineae*. Members of the genus *Corynebacterium* are characterized by producing short-chain C22 to C36 mycolic acids, also called corynomycolic acids, with simple chemical structure [57].

**Fig. 4:**
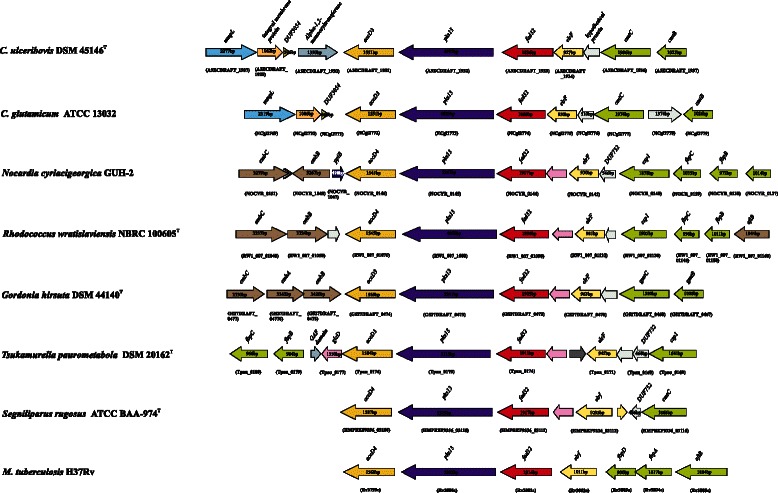
Cluster of genes encoding for enzymes involved in mycolic acid biosynthesis in *C. ulceribovis* DSM 45146^T^ and selected species of some relevant genera of *Corynebacterineae*. The cluster consists of at least five genes, all of them transcribed in the same direction. The *pks13*, *fadD32* and *accD3*/*accD4* othologs were found in all genomes examined and the genetic organiszation surrounding the *pks13* gene was highly conserved. Slight differences between the species examined is apparent in the mycolyltransferase (green colored) region. Orthologs are shown by matching colors

Examination of the genome of *C. ulceribovis* DSM 45146^T^ revealed the presence of homologs of genes encoding for proteins with known functions in the pathway of mycolic acids biosynthesis, processing and subsequent transport for deposition in the cell wall. These genes comprising: *accD3* encoding for an acyl-CoA carboxylase complex (A3ECDRAFT_1931), which catalyzes the carboxylation of palmitoyl-CoA to yield carboxylated intermediate [62–64]; *fadD32* encoding for an acyl-CoA synthetase/AMP ligase FadD32 (A3ECDRAFT_1933), which catalyzes the activation of the meromycolate chain through the formation of meroacyl-ADP before transfer to the polyketide synthase [64, 65]; *pks13* encoding for a polyketide synthase (A3ECDRAFT_1932) that performs the condensation of two fatty acids to form a 2-alkyl-3-keto mycolate precursor [66]; *elrF* encoding for the envelope lipid regulation factor ElrF (A3ECDRAFT_1934), _0314, _0659, _0660, _0935), which plays a role in the regulation of mycolic acid compositions in response to thermal variation in the environment [67]; *cmtA*, *cmtC* and *cmtB* encoding for trehalose mycolyltransferases (A3ECDRAFT_0077), (A3ECDRAFT_1936) and (A3ECDRAFT_1937), respectively, which catalyze: a) the transfer of mycolyl residue onto trehalose, thereby generating TMM, b) the transfer of one molecule TMM to another TMM leading to the formation of TDM, and c) the transfer of mycolate from TMM to arabinogalactan, forming the cell wall arabinogalactan-mycolate polymer [68–70]; *mmpL* encoding for membrane transport proteins of the MmpL family (A3ECDRAFT_0066, A3ECDRAFT_1927, A3ECDRAFT_2155) which is involved in the translocation of TMM to the outside of the bacterial cell for subsequent use as substrate for cell wall mycolylation [71]; and *cmrA* encoding for short-chain dehydrogenase/reductase CmrA (A3ECDRAFT_1367), the enzyme catalyzes the reduction of the mycolate precursor to produce the mature trehalose mycolates and subsequent covalent attachment onto the cell wall [72]. These genes clustered together forming a locus in the chromosome (Fig. 4). The overall organization of the entire locus in all mycolic acid-containing *Actinobacteria* is almost identical, although a slight difference is apparent in the mycolyltransferase region (Fig. 4). This gene repertoire is consistent with the detection of mycolic acids in the cell envelope of *C. ulceribovis* DSM 45146^T^ by thin-layer chromatography [1].

#### Phospholipids biosynthesis

Phospholipids are fundamental structural components of biological membranes that are also associated with energy production, nutrient uptake, protein export, and various sensing/signaling reactions in the living cells. The major phospholipids species found in members of the genus *Corynebacterium* are phosphatidylglycerol (PG), lysophosphatidylglycerol (LPG), cardiolipin (CL), phosphatidylinositol (PI) and phosphatidylinositol-dimannoside (PIM2) [73]. Thirteen genes related to phospholipid pathway were identified in the genome of *C. ulceribovis*. Among these were four genes encoding enzymes involved in phosphatidic acid (PA) biosynthesis via two pathways: two paralogs of *plsC* genes encoding for 1-acyl-sn-glycerol-3-phosphate acyltransferase PlsC ([EC:2.3.1.51]; A3ECDRAFT_1544 and A3ECDRAFT_1945), which catalyze the sequential addition of acyl moieties from acyl-CoA to sn-1 and sn-2 position of glycerol-3-phosphate to form phosphatidic acid [74]; two *dagk* paralogs encoding for diacylglycerol kinase DGK (A3ECDRAFT_0383 and A3ECDRAFT_0501), which catalyze the phosphorylation of diacylglycerol (DAG) to produce PA [75]. A single copy of the *cdsA* gene encoding for phosphatidate cytidylyltransferase CdsA ([EC:2.7.7.41], A3ECDRAFT_0714), which catalyzes the conversion of phosphatidic acid to cytidine diphosphate-diacylglycerol (CDP-DAG), a key intermediate in phospholipids biosynthesis [76]. Two genes encoding for proteins annotated as phosphatidylglycerophosphate synthase ([EC:2.7.8.5], A3ECDRAFT_0785) and phosphatidylglycerophosphate phosphatase ([EC:2.7.8.5], A3ECDRAFT_1077) required for the synthesis of PG. The first enzyme catalyzes the condensation of CDP-DAG with glycerol-3-phosphate yielding phosphatidylglycerophosphate (PGP), whereas the second enzyme catalyzes the subsequent dephosphorylation of PGP to produce PG. A copy of the *cls* gene encoding for cardiolipin synthase Cls ([EC:2.7.8.-], A3ECDRAFT_0953), which catalyzes the synthesis of cardiolipin through the condensation of two phosphatidylglycerol molecules [77]. A copy of the *pgsA* gene encoding for PI synthase PIS ([EC:2.7.8.5], A3ECDRAFT_0837), which catalyzes the synthesis of PI from CDP-DAG and myo-inositol [78]. The *pimA* and *pimB* genes encoding for phosphatidylinositol α-mannosyltransferase PimA ([EC:2.4.1.57], A3ECDRAFT_0839) and phosphatidylinositol α-1,6-mannosyltransferase PimB ([EC:2.4.1--], A3ECDRAFT_1542), respectively, which are involved in the mannosylation steps of PI to produce PIMs. PimA catalyzes the committed step in PIMs biosynthesis, transferring a mannose residue from GDP-Man to the 2-OH of the inositol ring of PI producing PIM1 [79, 80], whereas PimB catalyzes the transfer of a second Man residue from GDP-Man to the 6-position of the inositol moiety of PIM1 resulting in the formation of PIM2 [81–83]. Moreover, the genes *ino1* and *impA* encoding for *myo*-inositol-3-phosphate synthase Ino1 ([EC:5.5.1.4], A3ECDRAFT_2002) and inositol monophosphatase ImpA ([EC:3.1.3.25], A3ECDRAFT_0801 and A3ECDRAFT_1648), respectively, were present. Both Ino1 and ImpA enzymes are involved in the biosynthesis of *myo*-inositol from glucose-6-phosphate [84, 85].

**Fig. 5:**
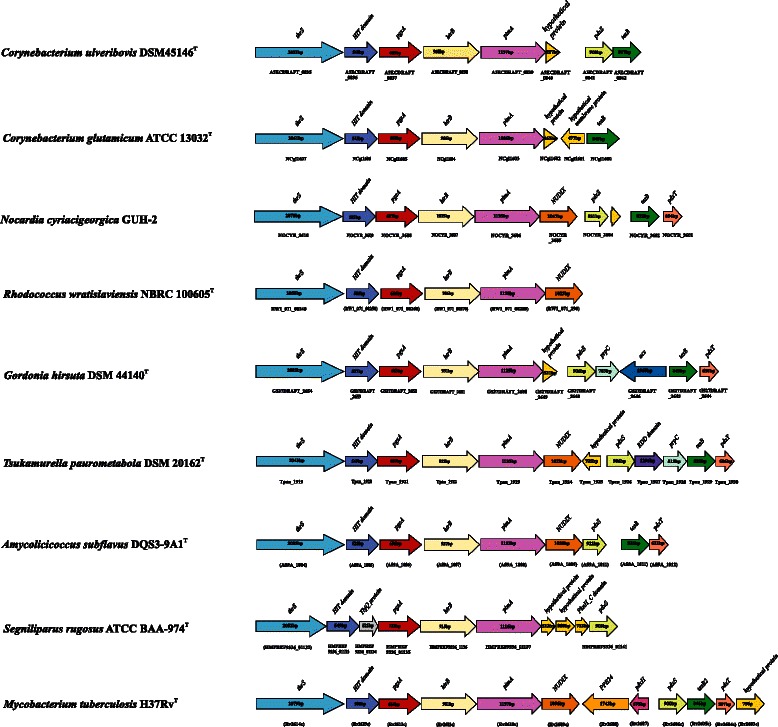
Comparison of the genetic organization of the *pgsA* and *pimA* genes in *C. ulceribovis* DSM 45246^T^ and PI/PIMs-producing species of some selected genera of *Corynebacterineae*. In all examined species the *pgsA, htrB* and *pimA* genes are colocalized confirming its link to PI and PIMs biosynthesis. Orthologs are shown by matching colors

Although the *pgsA* paralogs (A3ECDRAFT_0785, A3ECDRAFT_0837 and A3ECDRAFT_1077) were annotated as PgsA/CDP-diacylglycerol--glycerol-3-phosphate 3-phosphatidyltransferase, it seems likely that they are functionally not related. The *pgsA* (*A3ECDRAFT_0837*) genomic region in *C. ulceribovis* showed an organization similar to that found in other bacteria (Fig. 5). In all these groups *pgsA* is the second gene of a cluster of four to five genes potentially organized as an operon. The first ORF of this cluster (*A3ECDRAFT_0836*) located upstream of the *pgsA* gene encoded a protein of unknown function. The third ORF (*A3ECDRAFT_0838*) located downstream of the *pgsA* gene encoded a protein with similarities to bacterial acyltransferases (showed 53 % identity with homolog Rv2611c in *M. tuberculosis* H37Rv). The fourth ORF (*A3ECDRAFT_0839*) encoded a putative α-mannosyltransferase PimA (showed 49 % identity with homolog Rv2610c in *M. tuberculosis* H37Rv). Genetic evidences have showed that the *pimA* ortholog (*RV2610c*) in *M. tuberculosis* H37Rv encoded an essential enzyme for mycobacterial growth that initiates the biosynthetic pathway of PIMs [86, 87]. Therefore in *C. ulceribovis*, the presence of *pgsA* (*A3ECDRAFT_0837*) and *pimA* (*A3ECDRAFT_0839*) genes together within a cluster of genes suggested that PgsA (A3ECDRAFT_0837) may be a phosphatidylinositol synthase involved in PI biosynthesis which could be mannosylated by PimA (A3ECDRAFT_0839) leading to the synthesis of PIM. However, experimental verification of the function of the protein (A3ECDRAFT_0837) remains to be performed.

### Cell wall biogenesis and the penicillin binding proteins (PBPs)

**Fig. 6:**
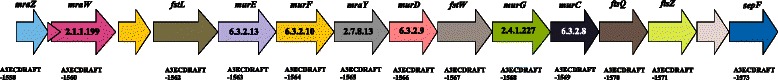
Division and cell wall (*dcw*) cluster in *C. ulceribovis* DSM 45246^T^. The region consists of a cluster of genes involved in cell division and peptidoglycan synthesis

*C. ulceribovis* possesses genes encoding for a complete set of enzymes involved in peptidoglycan (PG) biosynthesis, including MurABCDEFGI, alanine racemase ([EC:5.1.1.1], A3ECDRAFT_0263) and D-alanyl-D-alanine ligase ([EC:6.3.2.4]; A3ECDRAFT_0671). MurA, a UDP-GlcNAc enolpyruvyl transferase, catalyzes the first committed step of peptidoglycan synthesis by transferring an enolpyruvate from PEP to UDP-GlcNAc resulting in the formation of UDP-N-acetylglucosamine-enoylpyruvate, which subsequently reduced by MurB to UDP-N-acetylmuramic acid (UDP-MurNAc). The amino acid ligases MurC, MurD, MurE and MurF catalyze the successive addition of L-alanine, D-glutamic acid, *meso*-diaminopimelic (*meso*-DAP) or L-lysine and D-alanyl-D-alanine dipeptide, repectively, to the D-lactoyl group of UDP-MurNAc [88]. The translocase MraY catalyzes the transfer of MurNAc-pentapeptide motif from UDP-MurNAc-pentapeptide to an undecaprenyl phosphate carrier lipid anchored in the cytoplasmic membrane forming the first membrane linked intermediate (Lipid I). The MurG protein catalyzes the transfer of GlcNAc from UDP-GlcNAc onto lipid I, generating lipid II [89]. The *murCDEFG* genes were organized in cluster located in the center of conserved *dcw* (division cell wall) region in the order shown in (Fig. 6), whereas the *murABI* genes were located elswhere in the chromosome.

*meso*-DAP, the third residue in the PG pentapeptide [90], is an important chemotaxonomic marker of members of the *Corynebacterineae* including the genus *Corynebacterium* and it is essential for both peptidoglycan and lysine biosynthesis in bacteria. From genome sequencing data, it was clear that *C. ulceribovis* should synthesize *meso*-DAP from aspartate via the dehydrogenase variant of the DAP-pathway [91, 92]. The genes *dapA*, *dapB*, *dapC* and *dapD* are present in the genome, whereas the *dapE* and *dapF* genes are missing from the genome. Moreover, the *ask* (encoding aspartokinase [EC:2.7.2.4], (A3ECDRAFT_0021) and *asd* (encoding aspartate semialdehyde dehydrogenase [EC:1.2.1.11], A3ECDRAFT_0022), genes are found clustered in an operon. Aspartokinase and aspartate semialdehyde dehydrogenase catalyze the first two enzymatic steps of the DAP-pathway, leading to conversion of aspartate to aspartate semialdehyde. The *lysA* gene encoding for diaminopimelate decarboxylase ([EC:4.1.1.20] A3ECDRAFT_0568), which catalyzes the conversion of *meso*-DAP to L-lysine, is also present in the genome.

Furthermore, *C. ulceribovis* genome harbors seven genes encoding for penicillin binding proteins (PBPs) that catalyze the final steps of peptidoglycan synthesis. Two genes encoding for the bi-functional high molecular-mass (HMM) PBP of 1A family, designated PBP1A (A3ECDRAFT_2000) and PBP1B (A3ECDRAFT_0034), which have transpeptidase-transglycosylase activities and catalyze the elongation of the uncross-linked glycan chains of the PG. Three genes encoding for HMM PBP 2 family, designated PBP2A (A3ECDRAFT_2052), PBP2B [FstL] (A3ECDRAFT_1562) and PBP2C (A3ECDRAFT_0722), which contain transpeptidase domains and an additional dimerisation domains and believed to play a role in cell morphogenesis. Two genes encoding for low-molecular mass (LMM) PBP of type PBP4, designated DacB (A3ECDRAFT_1828), and one of type PBP 5/6, designated DacC ([EC:3.4.16.4]; A3ECDRAFT_0319). The Dac proteins are D,D-carboxypeptidases which catalyze the cleavage of the terminal D-Ala-D-Ala bond making the stem peptide unavailable for transpeptidation and through this activity regulate the amount of cross-linking that can occur within the PG [93].

Moreover, *C. ulceribovis* genome contains two *ldt* genes encoding two L, D-transpeptidases (Ldt), LdT1 (A3ECDRAFT_1351) and LdT2 (A3ECDRAFT_1870). The L,D-transpeptidases are a group of carbapenem sensitive enzymes that participate in the remodeling of the peptidoglycan network by formation of 3 → 3 cross-links between two adjacent *meso*-DAP residues (*meso*-Dap → *meso*-Dap bridges) instead of the 4 → 3 cross-links (D-Ala → *meso*-DAP) generated by the D,D-transpeptidase activity of the PBPs and can thus render the peptidoglycan resistant to the hydrolytic activity of endopeptidases [94, 95].

### Cofactor biosynthesis

Organic cofactors play crucial roles in the catalysis of biochemical reactions in the metabolism of all living organisms. Inspection of *C. ulceribovis* DSM 45146^T^ genome revealed the expression of enzymes involved in the *de novo* biosynthetic pathways for several cofactors such as pyridoxal-5-phosphate, lipoic acid, flavin nucleotides, folate, pantothenate, thiamine, nicotinic acids biotin and menaquinones.

The supplied additional files give an overview of the *de novo* biosynthetic and salvage pathways for some of these cofactors.

The genomes of *C. ulceribovis* DSM45146^T^ harbors only one gene encoding for a pyridoxal 5’-phosphate synthase PdxS ([EC:4.3.3.6], A3ECDRAFT_0841). Pyridoxal-5-phosphate seems to be synthesized in *C. ulceribovis* from ribulose 5-phosphate and a 3 carbon sugar via the deoxyxylulose 5-phosphate-independent pathway as named by [96]. The *lipA* and *lipB* genes encoding for lipoyl synthase LipA ([EC:2.8.1.8], A3ECDRAFT_1516) and lipoyl(octanoyl) transferase LipB ([EC:2.3.1.181], A3ECDRAFT_1517) that catalyze the two consecutive steps of lipoic acid *de novo* biosynthesis were identified.

#### Riboflavin (Vitamin B2) biosynthesis

Riboflavin is synthesized from guanosine 5’-triphosphate (GTP) and ribulose 5’-phosphate. It is an essential component of the basic metabolism, being the precursor of the flavin cofactors, flavin mononucleotide (FMN) and flavin adenine dinucleotide (FAD), which serve as prosthetic groups for many oxidoreductases [97]. The genome of *C. ulceribovis* has all genes encoding for the enzymes involved in riboflavin synthesis (Additional file 1). The *rib* operon encoding for a GTP cyclohydrolase II (RibA)/3,4-dihydroxy 2-butanone 4-phosphate synthase (RibB), diaminohydroxyphosphoribosylaminopyrimidine deaminase (RibD)/5-amino-6(5-phosphoribosyl-amino)uracil reductase (RibD), riboflavin synthase (RibE), and 6,7-dimetyl-8-ribityllumazine synthase (RibH). These enzymes form a pathway that yields one riboflavin molecule from one molecule of GTP and two molecules of ribulose 5-phosphate. At the next stage, the bifunctional riboflavin kinase/FAD-synthase converts riboflavin to FMN and FAD.

#### Folic acid (Vitamin B9) biosynthesis

Genes encoding for all the enzymes of the folate biosynthetic pathways are present (Additional file 2). The first enzyme of the pterin branch is GTP cyclohydrolase (FolE), which catalyzes the conversion of GTP to 7,8-dihydroneopterin triphosphate [98], which is converted to the corresponding monophosphate by alkaline phosphatase D [EC:3.1.3.1]. The three genes *folBKP*, which encode the three enzymes dihydroneopterin aldolase, 2-amino-4-hydrox-6-hydroxymethyldihydropteridine diphosphate and dihydropteroate synthase, respectively, formed an operon. The three enzymes catalyze the stepwise conversion of dihydroneopterin to 7,8-dihydropteroate. The resulting dihydropteroate is converted to dihydrofolate by dihydrofolate synthase [EC:6.3.2.12], which is reduced by dihydrofolate reductase [EC:1.5.1.3] to tetrahydrofolate.

#### Pantothenic acid (Vitamin B_5_) and coenzyme A (CoA) biosynthesis

Like other bacteria, *C. ulceribovis* synthesizes coenzyme A (CoA) via pantothenic acid from aspartate and α-ketoisovalerate (Additional file 3). The CoA biosynthetic route requires nine enzymes: four to synthesize pantothenic acid I-VI) and five to produce CoA (VI-XI). With the exception of the gene encoding for 2-dehydropantoate 2-reductase PanE (EC1.1.1.169), which catalyzes the reduction of 2-dehydropantoate (IV), all pantothenate and CoA biosynthesis genes are annotated in *C. ulceribovis*. Although the genome lacks the *panE* gene encoding for 2-dehydropantoate 2-reductase (KPR), a gene (*A3ECDRAFT_1818*) encoded for a predicted oxidoreductase, which contains short-chain dehydrogenase (SDR) and DUF2520 domains, was present in the genome. BLASTP analysis revealed that the protein (A3ECDRAFT_1818) was 41 % identical to ketopantoate reductase (PanE/ApbA) in *Corynebacterium durum* F0235. Homologs of this KPR protein are present in other bacterisa such as *Enterococcus faecalis* V583 (EF1861), *Francisella**novicidia* (FTT1388) and *Clostridium difficile*. The KPR protein has been shown to also catalyze the conversion of 2-dehydropantoate to pantoate in *Francisella* species [99].

In the *p*ABA (*para*-aminobenzoic acid) branch of the pathway, chorismate is aminated to 4-amino-deoxychorismate by para-aminobezoate synthase [EC:2.6.1.85] which subsequently converted to *p*ABA by aminodeoxychorismate lyase [EC:4.1.3.38]. The enzyme dihydropteroate synthase also catalyzes the condensation of *p*ABA with 6-hydroxymethyl 7,8-dihydropterin pyrophosphate yielding dihydropteroate.

#### Thiamine (Vitamin B1) biosyntheis

Thiamine in its active form, thiamine pyrophosphate, is an essential cofactor for several microbial enzymes of the carbohydrate metabolism [100]. The genes encoding proteins related to the biosynthesis of thiamine pyrophosphate are present in the genome of *C. ulceribovis* DSM 45146^T^. The thiamine biosynthetic pathway of *C. ulceribovis* 45146^T^ is outlined in (Additional file 4). Thiamine monophosphate (XIII) is formed by coupling of two independently synthesized moieties, 4-amino-2-methyl-5-β-hydroxyethyl thiazole phosphate (IX) and 4-amino-2-methyl-5-hydroxymethylpyrimidine pyrophosphate (XII). At the next step, thiamine monophosphate is phosphorylated by the enzyme ThiL to form thiamine pyrophosphate. The 4-amino-2-methyl-5-hydroxymethylpyrimidine pyrophosphate (XII) is produced from aminoimidazole ribotide (X), an intermediate of purine biosynthesis pathway [101]. Hydroxymethyl pyrimidine synthase (ThiC) and phosphomethylpyrimidine kinase (ThiD) catalyze the conversion of (X) to form (XII). In *C. ulceribovis* DSM 45146^T^, 4-amino-2-methyl-5-β-hydroxyethyl thiazole phosphate (IX) is derived from an oxidative condensation of cysteine, glyciene and 1-deoxy-D-xylulose 5-phosphate (DXP). Several genes such as *thiG, thiO, iscS,* and *Dxs* are involved in this process.

#### Nicotinic acid (Vitamin B3) and nicotinamide adenine dinucleotide NAD biosynthesis

NAD and its reduced and phosphorylated derivatives, NADH, NADP and NADPH, function as reducing equivalents for cellular biochemistry and energy metabolism. The genome of *C. ulceribovis* 45146^T^ carries the genes encoding for enzymes involved in NAD biosynthesis via both the canonical *de novo* pathway from L-aspartate and the salvage biosynthetic pathway from nicotinamide. In the *de novo* pathway, nicotinic acid mononucleotide (NaMN) is synthesized in three enzymatic steps from L-aspartate followed by two enzymatic steps to complete the synthesis of NAD (Additional file 5). In the salvage biosynthesis, nicotinamide is converted in a four-step pathway through nicotinate, nicotinate D-ribonucleotide and deamino NAD^+^ to intact NAD^+^ (Additional file 5).

#### Biotin (Vitamin H) biosynthesis

Biotin is an essential cofactor for biotin-dependent carboxylases, which catalyze the transfer of a carboxylate group from a donor to an acceptor molecule [102]. Biotin synthesis can be subdivided into the synthesis of pimeloyl-CoA from pimelic acid followed by the biotin ring assembly [103]. The *bioA-bioD* and *bioB* genes encoding for the enzymes involved in the biotin ring assembly were identified in *C. ulceribovis* DSM 45146^T^ genome. However, the pathway of biotin biosynthesis in *C. ulceribovis* DSM 45146^T^ is incomplete due to the lack of at least of the *bioF* and *bioW* genes. Moreover, the genome contains the *bioY-bioM-bioN* genes encoding for the protein components BioY (A3ECDRAFT_0764) -BioM (A3ECDRAFT_0763) –BioN (A3ECDRAFT_0762), which constitute tripartite biotin transporter [104]. The *birA* gene encoding for the BirA protein was also identified.

#### Menaquinone (Vitamin K2)

Menaquinone (MK) plays a key role as an electron carrier in the electron transport of the respiratory chain in prokaryotes [105]. The genome of *C. ulceribovis* is also equipped with the genes for the biosynthetic pathway of menaquinone from chorismate. In this pathway chorismate is converted into 1,4-dihydroxy-2-naphthoate (DHNA) via isochorismate by five enzymes encoded by the *menFDCEB* genes. DHNA is converted to MK after prenylation (catalyzed by MenA) and methylation (catalyzed by MenG). Since menaquinones are the only type of isoprenoid quinones found in the genera of the suborder *Corynebacterineae*, including the genus *Corynebacterium*, the presence of genes encoding for enzymes catalyzing the biosynthesis of menaquinone in the genome of *C. ulceribovis* DSM 45146^T^ is consistent with its classification in the genus *Corynebacterium*. Menaquinones are widely used as chemotaxonomic markers. The taxonomic value of menaquinones lies on their chain length and degree of unsaturation [106].

### CRISPR/Cas system and immunity to phage attack

Analysis of the genome sequence revealed that *C. ulceribovis* employs various defense mechanisms to overcome phage infections. These include restrisction of penetrating phage DNA (restriction-modification (R-M) system), abortive phage infection (Abi) system, and the clustered regularly interspaced short palindromic repeats (CRISPR)-associated (Cas) proteins (CRISPR/Cas) system.

The genome contains six paralogs of the *hsd* genes encoding for type I R-M enzymes. These include two *hdsR* paralogs encoding for two R subunits of type I restriction enzyme HsdR ([EC:3.1.21.3], A3ECDRAFT_1191 and A3ECDRAFT_1675), two *hsdM* paralogs encoding for two M subunits of type I restriction enzyme HsdM ([EC:2.1.1.72], A3ECDRAFT_1187 and A3ECDRAFT_1677) and two *hdsS* paralogs encoding for two S subunits of type I restriction enzyme HsdS ([EC:3.1.21.3], A3ECDRAFT_1188 and A3ECDRAFT_1676). The HsdR subunit is responsible for restriction, the HsdM subunit is involved in modification and the HsdS subunit is responsible for specific sequence recognition. None of them reveals any activity as a single protein [107]. For modification activity, a combination of one HsdS and two HsdM subunits is required and for restriction activity all subunits are absolutely required in a stoichiometric ratio of R_2_M_2_S_1_ [107]. The M_2_S_1_ multifunctional enzyme acts as protective methyltransferase [108], whereas the holoenzyme exhibits both endonucleolytic and helicase activities. The principal function of the R–M system is to protect the bacterial cell against invading DNA, including viruses [109].

In addition, a gene (*A3ECDRAFT_0290*) encoding a protein annotated as Abi-like protein was identified. This protein contains an Abi_2 domain (pfam07751), which has been shown to mediate bacteriophage resistance by abortive infection [110]. Activation of Abi protein limits phage replication within a bacterial population and promotes bacterial cell death [111, 112].

Moreover, *C. ulceribovis* DSM 45146^T^ genome contains two CRISPRs loci together with the associated *cas* genes. CRISPR locus 1 contains 1070 bp and harbors 17 spacer sequences and is not specified by the presence of *cas* genes in the direct proximity. CRISPR locus 2 contains 6893 bp and harbors 102 spacer sequences and is flanked by seven *cas* genes [*cas3* (*A3ECDRAFT_1586*), *cse1* (*A3ECDRAFT_1587*), *cse2* (*A3ECDRAFT_1588*), *cas*7 (*A3ECDRAFT_1589*), *cas*5 (*A3ECDRAFT_1590*), *cas*6 (*A3ECDRAFT_1591*) and *cas*1 (*A3ECDRAFT_1592*)]. The consesus sequences of the direct repeats of the two CRISPR regions are identical having a length of 28 bp (GTGTTCCCCGCGCAGGCGGGGATGAGCC) and separated by spacers with variable nucleotide sequences. CRISPRs provide the cell with aquired immunity to protect against bacteriophages, plasmids and other mobile genetic elements by a RNA interference-like mechanism [113, 114].

### Insight into protein secretion systems

Secreted proteins play essential roles in bacteria, including the colonization of niches and host-pathogen interactions [115, 116]. In Gram-positive bacteria proteins secretion is mediated mainly by the general secretory (Sec) and the twin-arginine translocation (Tat) pathways. Some Gram-positive bacteria e.g. mycobacteria, nocardia and corynebacteria have a specialized type VII secretion system (T7SS) for secretion of WXG100 family proteins.

Inspection of *C. ulceribovis* DSM 45146^T^ genome revealed the presence of all genes encoding proteins for the Sec translocation system. These include proteins forming the main membrane channel-forming complex SecYEG (A3ECDRAFT_0227/A3ECDRAFT_0157/A3ECDRAFT_0912), the cytosolic ATPase SecA (A3ECDRAFT_0372 and A3ECDRAFT_1078), the auxiliary proteins SecD (A3ECDRAFT_0848), SecF (A3ECDRAFT_0849) and YajC (A3ECDRAFT_0847), and the chaperones Ffh (A3ECDRAFT_0690) and FtsY (A3ECDRAFT_0689). As in other Gram-positive bacteria, the genome lacks homologs of the SecB protein, the chaperone that targets protein to the Sec translocon for passage through the cytoplasmic membranes. Genes encoding for the twin-arginine translocase (Tat) system, *tatA/E, tatB*, *tatC*, and *tatD* were also present in the genome. Like the majority of other sequenced actinobacterial genomes, the *tatA/E* gene (*A3ECDRAFT_0977*) was found next to *tatC* (*A3ECDRAFT_0978*), while the *tatB* gene (*A3ECDRAFT_0538*) and the *tatD* gene (*A3ECDRAFT_1228*) were separately located. The distinguishing feature of the TAT system is its ability to translocate fully folded proteins across the cytoplasmic membrane using the transmembrane proton gradient as the main driving force for translocation [117].

A putative type IVb pilus-encoding gene cluster, similar to the *tad* (tight adherence) locus in *Haemophilus actinomycetemcomitans*, was identified in the genome of *C. ulceribovis*. The genes of this *tad* locus appear to be organized as two adjacent clusters. The first cluster contained four genes encoding for: homolog of the TadZ protein (A3ECDRAFT_0049), followed by the TadA protein (A3ECDRAFT_0050), followed by two integral membrane proteins, TadB (A3ECDRAFT_0051) and TadC (A3ECDRAFT_0052). The second cluster contained three genes encoding for: a low-molecular weight protein (68 aa) containing DUF4244 domain (A3ECDRAFT_0053), followed by an unkown protein (A3ECDRAFT_0054), followed by a TadE-like protein (A3ECDRAFT_0055). Not linked to the *tad* locus, a gene encoding a putative prepilin peptidase PilD (A3ECDRAFT_0873), which was found located distantly in the genome. The *tad* export apparatus facilitates the export and assembly of pili, which mediate the nonspecific adhesion of bacteria to surfaces and are essential for host colonization and pathogenesis [118–120].

**Fig. 7:**
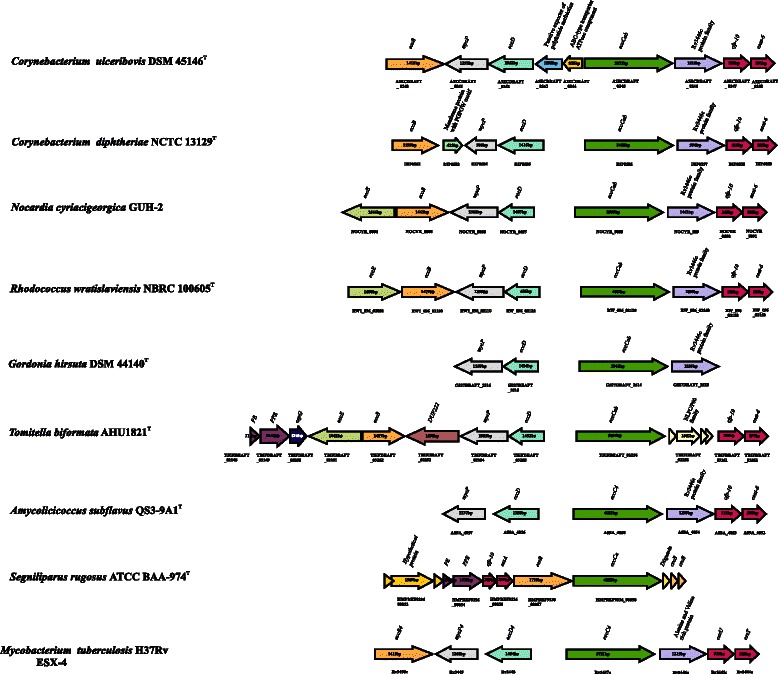
Comparison of gene clusters that encode type VII secretion system (T7SS also ESX) in *C. ulceribovis* DSM 45146^T^ and variants that are present in other mycolic acid-containing taxa of the *Corynebacterineae*. Six genes encoding for six proteins are generally present in all the examined species. These proteins are: two members of the ESAT-6 family (Esat-6 and CFP-10); a member of the FtsK/SpoIIIE family (EccCab); a subtilisin-like protease (MycP); an integral membrane protein with 10–11 transmembrane domains (EccD); a member of another membrane-protein family (EccB); In addition, two proteins the PE (proline-glutamine) and PPE (proline-proline-glutamine) encoded by two genes are shared by some, but not all T7SS systems. Orthologs are shown by matching colors

In addition, genes encoding proteins for type VII secretion system (ESX/T7SS) were also present in *C. ulceribovis* DSM 45146^T^ genome. A region, comparable to region 4 (ESX-4) of *M. tuberculosis* H37Rv, containig nine genes (*A3ECDRAFT_0240- A3ECDRAFT_0248*) was identified (Fig. 7). The proteins encoded by these genes include: (A3ECDRAFT_0248), a WXG100/ESAT-6-like protein composed of 96 amino acids; (A3ECDRAFT_0247), a WXG100/CFP-10-like protein composed of 104 amino acids; (A3ECDRAFT_0246), Rv3446 potein family, C-terminal domain (alanine and valine rich protein); (A3ECDRAFT_0245), an ATPase with FtsK-SpoIIIE domain (EccCab); (A3ECDRAFT_0244), a putative ABC-type transporter-ATPase component; (A3ECDRAFT_0243), a putative exporter of polyketide antibiotics; (A3ECDRAFT_0242), an integral membrane protein with transmembrane helix regions (EccD); (A3ECDRAFT_0241), a subtilisin-like serine protease (MycP); (A3ECDRAFT_0240), a transmembrane protein (EccB). ESAT-6 (product of *esxA*) and CFP-10 (product of *esxB*) are members of a protein family that is characterized by a length of ~100 amino acid residues, containing a WXG motif and lack a distinguishable Sec-signal sequence [121]. Both of these proteins are important T-cell antigenic targets and are essential for the virulence of *M. tuberculosis* and *Staphylococcus aureus* [122, 123]. Type VII secretion system affects a range of bacterial processes including sporulation, conjugation and cell wall stability [124, 125].

## Conclusions

The availability of high-quality genome sequence from *C. ulceribovis* provided crucial insights into the broad biological functions of this organism. Genome analysis showed that the overall features of *C. ulceribovis* are similar to those of the genus *Corynebacterium*; it possesses a complete set of peptidoglycan biosynthesis genes, synthesizes *meso*-DAP from aspartate via the dehydrogenase pathway, possesses all genes for menaquinone biosynthesis from corismate and has complete set of genes for the biosynthesis and processing of mycolic acids. *C. ulceribovis* also possesses a single *fas1* gene encoding type I fatty acid synthase FAS I for *de novo* fatty acids biosynthesis and a complete set of genes associated with fatty acid degradation by the β-oxidation pathway. Genes encoding enzymes associated with the central carbohydrates metabolism were identified. *C. ulceribovis* possesses a complete TCA cycle and glyoxylate shunt; a functional PPP for generation of pentoses and NADPH for anabolic purposes; all gene necessary for glycogen metabolism; trehalose synthesis via the OtsA-OtsB pathway. The genome also contains genes encoding *myo*-inositol- 3-phosphate synthase and inositol monophosphatase involved in the biosynthesis of *myo*-inositol from glucose-6-phosphate as well as gene encoding for α-mannosyltransferase PimA leading to the synthesis of PIM. To meet cofactor requirements, several genes encoding for enzymes that catalyze *de novo* biosynthetic pathways for several cofactors are present in the genome. Finally the genome of *C. ulceribovis* harbors genes encoding proteins that protect the cells against the danger of bacteriophage infections. These include type I restriction enzymes (R-M enzymes), Abi-like protein that mediate bacteriophage resistance by abortive infection (Abi system) and CRISPER/cas system that serve as molecular “vaccination cards”.

## Additional files

Additional file 1:
**Biosynthesis of riboflavin and FAD.** I: Guanosine 5’-triphosphate; II: 2,5-Diamino-6-(5-phospho-D-ribosylamino)pyrimidin-4(3H)-one; III: 5-Amino-6-(5’-phosphoribosylamino)uracil; IV: 5-Amino-6-(5’-phospho-D-ribitylamino)uracil; V: 5-Amino-6-(1-D-ribitylamino)uracil; VI: D-Ribulose 5-phosphate; VII: 2-Hyroxy-3-oxobutyl phosphate; VIII: 6,7-Dimethyl-8-(D-ribityl)lumazine; IX: Riboflavin; X: Flavin mononucleotide; XI: Flavin adenine dinucleotide; [EC:3.5.4.25]: GTP cyclohydrolase II; [EC:3.5.4.26]: diaminohydroxyphosphoribosylaminopyrimidine deaminase; [EC:1.1.1.193]: 5-amino-6-(5-phosphoribosylamino)uracil reductase; [EC:3.1.3.-]: phosphoric monoester hydrolases; [EC:4.1.99.12]: 3,4-dihydroxy 2-butanone 4-phosphate synthase; [EC:2.5.1.78]: 6,7-dimethyl-8-ribityllumazine synthase; [EC:2.5.1.9]: riboflavin synthase; [EC:2.7.1.26]: riboflavin kinase; [EC:2.7.7.2]: FAD synthase. (EPS 228 kb) Additional file 2:
**Biosyntheis of folate.** I: GTP; II: 7,8-Dihydroneopterin 3’-triphosphate; III: Dihyroneopterin phosphate; IV: 2-Amino-4-hydroxymethyl-7,8-dihydropteridine; V: 2-Amino-4-hydroxy-6-hydromethyl-7,8-dihydropteridine-diphosphate; VI: 4-Aminobenzoate; VII: 7,8-Dihydropteroate; VIII: L-Glutamate; IX: 7,8-Dihydrofolate; X: Tetrahydrofolate; XI: Folate; XII: 4-Aminodeoxychorismate; XIII: Chorismate; [EC:3.5.4.16]: GTP cyclohydrolase I; [EC:3.1.3.1]: alkaline phosphatase D; [EC:4.1.2.25]: dihydropterin aldolase; [EC:2.7.6.3]: 2-amino-4-hydroxy-6-hydroxymethyldihydropteridine diphosphate; [EC:2.5.1.15]: dihydropteroate synthase; [EC:6.3.2.12]: dihydrofolate synthase; [EC:6.3.2.17]: tetrahydrofolate synthase; [EC:1.5.1.3]: dihydrofolate reductase; [EC:2.6.1.85]: para-aminobenzoate synthase; [EC:4.1.3.38]: 4-amino-4-deoxychorismate lyase. (EPS 381 kb) Additional file 3:
**Biosynthesis of pantothenate and coenzyme A.** I: Aspartate; II: β-Alanine; III: 2-oxoisovalerate; IV: 2-Dehydropantoate; V: Pantoate; VI: Pantothenate; VII: (R)-4’-Phosphothenate; VIII: (R)-4’-Phosphothenoyl-L-cysteine; IX: 4’-phospho-pantetheine; X: Dephospho-CoA; XI: Coenzyme A; [EC:4.1.1.11]: aspartate 1-decarboxylase; [EC:2.1.2.11]: 3-methyl-2-oxobutanoatehydroxymethyltransferase; [EC:1.1.1.169]: ketopantoate reductase; [EC:unkown]: unkown KPR; [EC:6.3.2.1]: pantoate--beta-alanine ligase; [EC:2.7.1.33]: pantothenate kinase; [EC:6.3.2.5]: phosphopantothenate--cysteine ligase; [EC:4.1.1.36]: phosphopantothenoylcysteine decarboxylase; [EC:2.7.7.3]: pantetheine-phosphate adenyltransferase; [EC:2.7.1.24]: dephospho-CoA kinase. (EPS 281 kb) Additional file 4:
**Biosynthesis of thiamine pyrophosphate.** I: L-Cysteine; II: [IscS]-SH; III: [IscS]-SSH; IV: Glycine; V: Iminoglycine; VI: Glyceraldehyde-3-phosphate; VII: Pyruvate; VIII: 1-Deoxy-D-xylulose-5-phosphate; IX: 4-methyl-5-β-hydroxyethyl thiazole phosphate; X: 1-(5’-Phopho-ribosyl)-5-aminoimidazole; XI: 4-Amino-2methyl-5-phosphomethylpyrimidine; XII: 4-amino-2-methyl-5-hydroxy-methylpyrimidine pyrophosphate; XIII: Thiamine monophosphate; XIV: Thiamine pyrophosphate;; [EC:2.8.1.7]: cysteine desulfurase; [EC:2.8.1.10]: thiazole synthase; [EC:1.4.3.19]: glycine oxidase; [EC:2.2.1.7]: 1-deoxy-D-xylulose-5-phosphate synthase; [EC:4.1.99.17]: phosphomethylpyrimidine synthase; [EC:2.7.4.7]: phosphomethylpyrimidine kinase; [EC:2.5.1.3]: thiamine-phosphate pyrophosphorylase; [EC:2.7.4.16]: thiamine-phosphate kinase. (EPS 340 kb) Additional file 5:
**Biosynthesis of nicotinic acid and nicotinamide nucleotides NAD.** I: L-Aspartate; II: Glycerone-phosphate; III: Iminoaspartate; IV: Quinolinate; V: Nicotinate D-ribonucleotide; VI: Deamino-NAD^+^; VII: Nicotinamide adenine dinucleotide; VIII: Nicotinamide adenine dinucleotide phosphate; IX: Nicotinate; X: Nicotinamide; [EC:1.4.3.16]: L-aspartate oxidase; [EC:2.5.1.72]: quinolinate synthase; [EC:2.4.2.19]: nicotinate-nucleotide pyrophosphorylase (decarboxylating); [EC:2.7.7.18]: nicotinate-nucleotide adenylyltransferase; [EC:6.3.1.5]: NAD^+^ synthase; [EC:2.7.1.23]: NAD^+^ kinase; [EC:2.4.2.1]: purine-nucleoside phosphorylase; [EC:3.5.1.19]: nicotinamidase. (EPS 291 kb)

## Abbreviations

KMG-I (project): The one thousand microbial genomes project
GEBA: Genomic Encyclopedia of *Bacteria* and *Archaea*
EC: Enzyme commission number
aa: Amino acids
MK: Menaquinone
*meso*-DAP: *meso*-diaminopimelic acid
PBP: Penicillin binding protein
HMM: High molecular mass
LMM: Low molecular mass
PIMs: Phoaphatidylinositol mannosides
TDM: Trehalose dimycolate
TMM: Trehalose monomycolate
ESAT-6: Early secreted antigen 6 kDa
CFP-10: Culture filtrate protein of 10 kDa
R-M: Restriction-modification system
